# Regular home use of dual-light photodynamic therapy as an adjunct to non-surgical periodontal treatment in smokers: a single-center randomized controlled clinical trial

**DOI:** 10.1007/s00784-025-06600-1

**Published:** 2025-11-06

**Authors:** Chrysoula Vakaki, Ioannis Fragkioudakis, Tommi Pätilä, Dimitra Sakellari

**Affiliations:** 1 Department of Preventive Dentistry, Periodontology and Implant Biology, School of Dentistry, Aristotle University of Thessaloniki, Thessaloniki, Greece; 2 Metropolia University of Applied Sciences, Helsinki, Finland

**Keywords:** Periodontitis, Smoking, Photodynamic Therapy, Adjunctive Treatment, Non-surgical Periodontal Therapy

## Abstract

**Objectives:**

This RCT assessed the effectiveness of daily home-applied dual-light antimicrobial photodynamic therapy (aPDT) as an adjunct to non-surgical periodontal therapy (NSPT) in smokers with Stage III or IV periodontitis.

**Materials and methods:**

Sixty smokers were randomized to receive either NSPT alone (control) or NSPT with daily for four months at-home application of aPDT, prior to oral hygiene (test group). Clinical parameters—bleeding on probing (BoP), probing depth (PD), clinical attachment loss (CAL), gingival recession (REC), full-mouth plaque score (FMPS), and Turesky Index—were assessed at baseline, 2 weeks, 4 months, and 6 months. aMMP-8 levels and patient-reported outcomes were also recorded.

**Results:**

Both groups demonstrated significant improvements in BoP, PD, and CAL (p < 0.05), but no significant intergroup differences were observed. BoP reduction at 2 weeks favored the test group (− 33.60% vs − 23.94%; *p* = 0.056). BoP continued to decrease at 4 and 6 months, reaching 29.55 ± 19.86% in the control group and 27.57 ± 16.43% in the test group at 6 months. The difference in mean BoP between the two groups at 6 months was not statistically significant (*p* = 0.680, Independent Samples Mann Whitney U Test). Plaque indices and aMMP-8 levels showed no significant differences. Compliance to dual-light aPDT averaged 77.7%; 72% of test participants were compliant. Greater compliance correlated with statistically significant improvements in mean values of all clinical parameters, whereas non-compliant patients presented with no changes (Related samples Friedman’s Two-Way Analysis). Patient-centered outcomes revealed positive feedback, with 84% of the patients willing to recommend the device and minor adverse effects.

**Conclusion:**

In smokers with advanced periodontitis, adjunctive home use of dual-light aPDT did not result in statistically significant improvements over NSPT alone in clinical parameters such as BoP, PD, or CAL. Future placebo-controlled, long-term clinical trials are warranted to further assess its potential role in supportive periodontal care.

**Clinical relevance:**

Within the limitations of this study, dual-light aPDT cannot be recommended as a superior adjunct to conventional treatment.

Trial registration: ClinicalTrials.gov Identifier: NCT05962801.

**Supplementary Information:**

The online version contains supplementary material available at 10.1007/s00784-025-06600-1.

## Introduction

Periodontal disease remains one of the most prevalent chronic conditions worldwide, and smoking is a major risk factor that substantially increases its prevalence and severity. Epidemiological studies show that smokers have approximately a two- to three-fold higher risk of periodontitis compared with non-smokers, and they generally experience poorer treatment outcomes [1]. These challenges highlight the need for adjunctive strategies to improve non-surgical periodontal therapy in smokers.

Antimicrobial photodynamic therapy (aPDT) has emerged as a promising adjunctive approach that relies on the interaction between a light source, a photosensitizer (PS), and molecular oxygen to generate reactive oxygen species (ROS) that can directly destroy microorganisms [2–4]. While in vitro studies demonstrate potent antimicrobial effects, clinical trials and systematic reviews have yielded mixed results, often reporting limited benefits when aPDT is used adjunctively with subgingival instrumentation [5–8]. These inconsistencies may arise from heterogeneity in light sources (for instance, laser parameters), types of photostimulable substances (PS), application protocols, and a lack of adequate consideration of host-related factors, such as smoking. Smokers are mostly excluded by studies evaluating the effects of aPDT due to detrimental effect of smoking on mitochondrial function. However, studies including smokers report contradictory results of aPDT to NSPT in smokers [9, 10].

Attention has recently shifted to antimicrobial blue light (aBL), which uses bacterial chromophores to induce oxidative damage. Black-pigmented bacteria, like *Porphyromonas gingivalis*, are particularly sensitive to aBL without exogenous photosensitizers [11, 12]. While aBL shows potential in planktonic cultures, mature biofilms are less susceptible [13], leading to interest in combined modalities. Dual-light aPDT, using aBL (405 nm) and near-infrared light (810 nm) with indocyanine green (ICG) as a photosensitizer, has shown superior antimicrobial effects in vitro and early clinical investigations [14, 15, 16–18]. This approach can counter bacterial resistance to single-wavelength irradiation and may disrupt mature biofilms effectively [14, 16]. Preliminary studies, including split-mouth and case reports, indicate reductions in plaque, inflammation, and pathogenic bacteria with regular use of the CE-marked dual-light device (Lumoral®) [19, 17, 18, 20]. However, these studies mainly focused on healthy individuals or those with mild to moderate periodontitis.While smoking cessation programs remain the most effective intervention for improving periodontal outcomes in smokers [21, 22], adherence to cessation is often low in real-world settings. Dual-light aPDT may therefore represent a supportive adjunct, as its dual-wavelength antimicrobial action can target smoking-associated pathogens and allow frequent at-home disruption of biofilm between professional sessions. This trial was designed to investigate whether such an adjunct could enhance NSPT outcomes in smokers who continue their habit, while acknowledging that cessation remains the primary therapeutic goal.

To date, no clinical trial has specifically assessed the adjunctive use of regular home-applied dual-light aPDT in smokers with severe periodontitis. Smoking is a well-established risk factor that alters host response and hampers treatment outcomes, further underscoring the need for novel adjunctive interventions in this population.

This single-center, randomized controlled clinical trial aimed to evaluate the clinical and biological effects of regular, home-applied dual-light aPDT as an adjunct to NSPT in smokers with Stage III–IV periodontitis.

The null hypothesis of the study was that dual-light aPDT does not provide additional clinical benefits to NSPT in smokers with Stage III–IV periodontitis.

## Materials and methods

### Study design

This study was a single-center, two-arm, parallel-group, randomized controlled clinical trial (RCT) evaluating the adjunctive efficacy of regular home use of dual-light antimicrobial photodynamic therapy (aPDT) with non-surgical periodontal therapy (NSPT) in adult smokers with Stage III or IV periodontitis. The trial was registered at ClinicalTrials.gov (NCT05962801), approved by the Ethics Committee of the School of Dentistry, Aristotle University of Thessaloniki (Approval No. 176/30–11–2022), and conducted according to the Declaration of Helsinki and ISO 14155 Good Clinical Practice standards. The study was conducted from January 2023 to June 2024, as also reflected in the CONSORT checklist. The primary outcome was the percentage change in bleeding on probing (BoP) from baseline to follow-up at 2 weeks, 4 months, and 6 months. Secondary outcomes included changes in probing depth (PD), clinical attachment loss (CAL), plaque indices (FMPS and Turesky), gingival recession (REC), and aMMP-8 levels.

Sixty participants were enrolled and randomized in a 1:1 ratio to test or control groups, as specified in the trial protocol (ClinicalTrials.gov Identifier: NCT05962801). Participants were assigned via simple randomization using a computer-generated number generator (www.randomizer.org): even numbers to the control group and odd numbers to the test group. Allocation was concealed in sealed envelopes, opened at baseline after clinical examination. A clinician not involved in treatment (D.S.) performed randomization. The test group received standard NSPT and daily home use of dual-light aPDT for four months; the control group received NSPT alone. Outcomes were evaluated at baseline, 2 weeks, 4 months, and 6 months, and compliance was assessed. The trial adhered to CONSORT 2010 guidelines (Suppl. Table 1).

### Sample size

Based on previously published data from dual-light aPDT studies, a power analysis was performed using SAS 9.4 (Cary, NC, USA). A randomized, split-mouth study by Nikinmaa et al. (2021b) [19] demonstrated significant plaque reduction in 15 patients, while in vitro work [15] indicated that the improved dual-light treatment protocol is approximately 3 log more potent. Additionally, a peri-implantitis pilot study [17] observed a significant reduction in plaque after 4 weeks in 7 patients. Based on this evidence, a sample size of 25 patients per group was estimated to detect a minimum 10% difference in BoP reduction (SD = 15%), with 80% power and α = 0.05. To account for potential dropouts, 30 patients were enrolled in each group.

### Eligibility criteria for study participants

Subjects over 18 years old referred to the undergraduate or postgraduate clinic of the department Preventive Dentistry, Periodontology, and Implant Biology from January 2023 to June 2024 to receive periodontal treatment through the appointment system of the School of Dentistry, Aristotle University of Thessaloniki, were eligible for recruitment according to the following inclusion and exclusion criteria.

Inclusion criteria

1. Periodontal disease stage III-IV, according to criteria of the 2017 World Workshop on the Classification of Periodontal and Peri-Implant Diseases and Conditions, with at least six sites with probing depth (PD) and clinical attachment loss (CAL) ≥ 5 mm and bleeding on probing (BoP) ≥ 15 teeth
2. Age of ≥ 18 years.
3. Smokers smoking ≥ 10 conventional cigarettes per day.
4. Agreement to participate in the study and to sign a written consent form.
5. Patients with ≥ 20 teeth.

Exclusion criteria

1. Patients with known allergies to indocyanine.
2. Patients with active carious lesions and removable dentures.
3. Need for prophylactic antimicrobial coverage.
4. Subgingival instrumentation in the previous 6 months.
5. Non-smoking status, e-smoking or smoking less than 10 cigarettes per day.
6. Antimicrobial therapy in the previous 6 months.
7. Immunomodifying conditions/diseases (e.g., diabetes mellitus, rheumatoid arthritis, osteoporosis).
8. Long-term use of medication that could interfere with periodontal response (e.g., bisphosphonates or calcium channel blockers).
9. Pregnancy or lactation.

### Intra-examiner reproducibility

All clinical measurements were conducted by one examiner (C.V.). Using a manual periodontal probe (CP-15, HuFriedy, Chicago, IL, USA), the examiner performed a full-mouth periodontal charting at six sites per tooth in three patients over two sessions, with a maximum 48-h interval. Calibration was acceptable if the baseline and 48-h measurements matched within > 85%. The examiner was not blinded to patients’ treatment status.

### Clinical examination

Clinical measurements were taken at six sites per tooth (mesiobuccal, buccal, distobuccal, mesiolingual, lingual, distolingual) using a CP-15 manual periodontal probe (HuFriedy, Chicago, IL, USA). The primary outcome was bleeding on probing (BoP), defined as bleeding within 15 s of gentle probing, expressed as the percentage of BoP-positive sites per patient.

Secondary parameters included probing depth (PD, mm), measured from the gingival margin to the base of the pocket, and clinical attachment loss (CAL, mm), measured from the cemento-enamel junction (CEJ) to the base of the pocket. CAL was calculated based on gingival margin position as follows: CAL = PD + gingival recession (REC) (when the gingival margin was apical to the CEJ); CAL = PD (when the margin was at the CEJ); and CAL = PD − coronal displacement (when the margin was coronal to the CEJ). Plaque was assessed using the Full-Mouth Plaque Score (FMPS) from O’Leary et al. (1972) [23], using dichotomous scoring (1 = plaque; 0 = no plaque) at six sites per tooth. The Turesky modification of the Quigley-Hein Index [24] was used on the buccal surfaces of upper and lower anterior teeth after erythrosine staining, with scores from 0 (no plaque) to 5 (≥ two-thirds crown coverage). The final score was the mean of all buccal anterior surfaces.

### Baseline examination and sampling

At the screening visit, participants received verbal and written information about the study. Those providing informed consent were scheduled for a second appointment for baseline data collection and randomization.

Clinical examination included full-mouth periodontal charting and anterior plaque assessment using the Turesky Index post-disclosure. Active-matrix metalloproteinase-8 (aMMP-8) levels in oral rinse were measured using the chairside Periosafe® test and read via the Oralyzer® digital reader (Dentognostics GmbH, Jena, Germany), following manufacturer guidelines.

### Initial periodontal therapy (Step 1)

All participants received individualized oral hygiene instruction and smoking cessation counseling. Professional supragingival debridement was performed using ultrasonic scalers and air-polishing (AIRFLOW® Prophylaxis Master, EMS, Bern, Switzerland). Participants were provided with an electric toothbrush (Jordan TB200B) and customized interdental brushes.

The test group additionally used the Lumoral® dual-light antimicrobial photodynamic therapy (aPDT) system daily. The device delivers simultaneous 405 nm blue and 810 nm NIR light, in combination with Lumorinse® (250 μg/mL indocyanine green). Participants were instructed to apply the device nightly for 10 min before oral hygiene, over four months. Compliance was monitored using a daily log (Suppl. Figure 2) and test group patients classified as ‘’compliant’’, having used the device for more than 70% of the expected days (over 84 of 120 days), whereas those below this threshold were considered “non-compliant.”

### Smoking cessation motivation

Smoking cessation encouragement through positive reinforcement approach was provided by the treating periodontist at baseline appointment. This included a fifteen minute- verbal information about the benefitial effect of smoking cessation on periodontal health and periodontal treatment outcomes. No standardized framework (e.g., 5A’s) was applied for this purpose and no printed materials or referrals were given. Feedback on smoking status was collected as self-reported cigarettes per day at each visit; no biochemical validation was performed. Patients were re-motivated on smoking quitting shortly at the beginning of every follow-up visit. and reinforced during follow-up visits.

### Early reassessment (Week 2)

Two weeks post-baseline, clinical re-evaluation was performed to assess initial response and compliance. BoP and the Turesky Index were reassessed, and aMMP-8 levels were remeasured using Periosafe/Oralyzer.

### Subgingival instrumentation (Step 2)

Following the 2-week assessment, patients underwent subgingival instrumentation of sites with a periodontal pocket depth (PD) of ≥ 4 mm, performed over two sessions within one week by a single clinician (C.V.). Ultrasonic debridement (AIRFLOW® Tip PS) was complemented with manual scaling (Gracey and Mini Five curettes; HuFriedy). No adjunctive antimicrobials or antiseptics were used.

### Re-evaluation (Month 4)

Three months after Step 2- and four months post-baseline, a comprehensive re-evaluation was conducted, which included full-mouth charting, Turesky Index and aMMP-8 testing. Sites with PD ≥ 5 mm were re-instrumented. Test group participants discontinued Lumoral® use. A structured 9-item Oral Health Quality of Life (OHQL) questionnaire assessed patient-reported outcomes (Suppl. Figure 3).

Between the subgingival debridement sessions and the 4-month reevaluation (3-month time interval), no additional supragingival cleaning or formal oral hygiene reinstruction sessions were provided. Patients were instructed to continue their individualized home-care routines (toothbrushing and interdental cleaning) without interim reinforcement. This protocol was chosen to minimize confounding professional influences on plaque control and to better isolate the adjunctive effects of dual-light aPDT.

### Final examination (Month 6)

Clinical parameters (PD, CAL, REC, BoP, FMPS), Turesky Index, aMMP-8 levels, and subgingival plaque samples were recorded. Patients achieving PD ≤ 4 mm, no BoP, and FM BoP < 30% were enrolled in a supportive periodontal care program, following current recommendations [25]. Others with acceptable hygiene were scheduled for further therapy. Patients were stratified by oral hygiene using a full-mouth plaque score (FMPS) threshold of 40%. While many guidelines suggest lower values (e.g., 20–25%) for optimal plaque control, thresholds up to 40% are commonly employed in clinical studies when classifying acceptable versus inadequate oral hygiene [26]. We selected the 40% cutoff as a pragmatic balance—stringent enough to reflect moderate control, yet sensitive to variability in patient adherence—facilitating meaningful subgroup analyses within our study population.

### Statistical analysis

Descriptive statistical indices, such as mean and standard deviation, were calculated for an initial examination of the data. Non-parametric tests, including the Kruskal–Wallis, Mann–Whitney U test, and Friedman’s Two-Way Analysis, evaluated any significant differences between or within suggested categories. The Z-test for column proportion with Bonferroni correction was applied to examine differences in parameter distribution among categories. The significance level remained at 0.05 throughout. All analyses used SPSS v29 software (IBM, SPSS Inc., USA).

## Results

### Demographic characteristics of the patient population

**Fig. 1:**
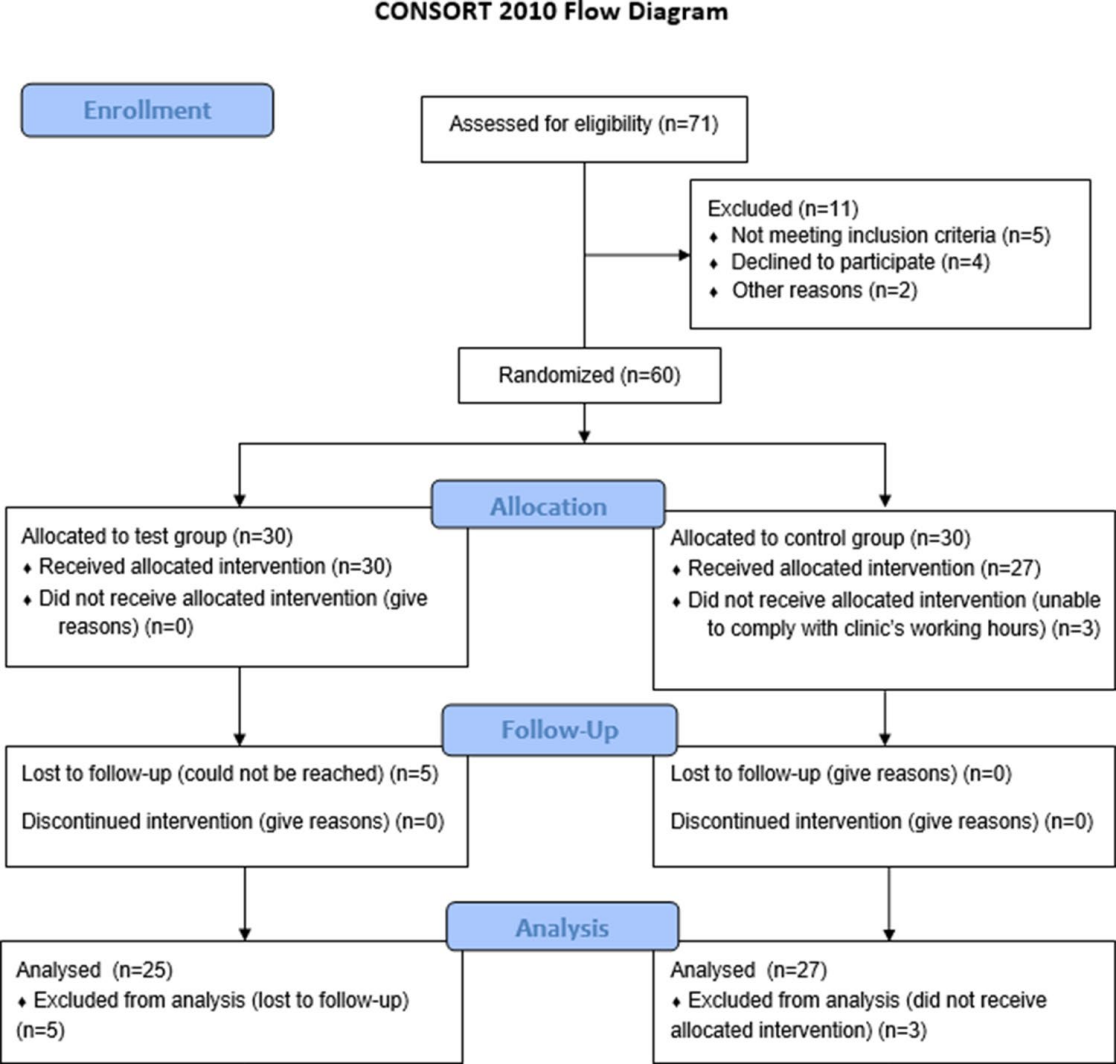
Flow diagram according to the CONSORT protocol for reporting Randomized Clinical Trials

Figure 1 displays the CONSORT flow diagram, which reports the number of patients assessed, enrolled, treated, and finally analyzed.

Table 1 outlines the sample characteristics from the trial. In the test group, 18 out of 25 patients and in the control group, 15 out of 27 were female. The control group's mean age was 57.27 (range, 34–71), while the test group's mean age was 50.56 (range, 28–68). The pack-years history, calculated based on daily smoking over the years [27], was self-reported at the baseline appointment. Mean pack-years were 28.02 (range 5.5–70) for the test group and 37.7 (range 5.6–94.5) for the control group.

**Table 1:** Patient characteristics

	Test group (25)	Control group (27)	p-value
Gender (F/M)	18/7	15/12	< 0.05*
Mean age ± SD (years)	50.56 ± 8.99	57.27 ± 7.55	0.05*
Smoking habits ± SD (pack years)	28.02 ± 13.83	37.7 ± 23.20	0.07

Abbreviations: *F* female, *M* male, *SD* standard deviation.

### Bleeding on probing (primary outcome)

BoP significantly decreased in both groups over time. At baseline, BoP levels were comparable (*p* = 0.728). By 2 weeks, both groups showed significant reductions, with a greater decrease in the test group that approached statistical significance (*p* = 0.056, Table 2). At 4 and 6 months, BoP remained significantly lower than baseline in both groups, with no significant between-group differences. Overall, both groups achieved similar BoP reductions by 6 months, indicating comparable clinical improvement.

**Table 2:** Clinical and biomarker outcomes over time (Baseline, 2 weeks, 4 months, 6 months)

Parameter	Group (n)	Baseline	2 weeks	4 months	6 months	Δ BL–2w	Δ BL–4 m	Δ BL–6 m	*p* (within)	*p* (between)
BoP (% sites)	Control (27)	59.09 ± 20.56	35.15 ± 20.21	28.30 ± 18.13	29.55 ± 19.86	–23.94 ± 21.94	–30.79 ± 25.43	–29.55 ± 29.52	< 0.001*	ns (*p* = 0.728 BL, 0.815 6 m)
	Test (25)	60.72 ± 24.16	27.12 ± 16.32	26.15 ± 16.24	27.57 ± 16.43	–33.60 ± 17.56	–34.57 ± 20.39	–32.35 ± 25.93	< 0.001*	0.056 (2w)
PD (mm)	Control (27)	3.97 ± 0.56	–	3.39 ± 0.48	3.35 ± 0.54	–	–0.58 ± 0.50	–0.62 ± 0.55	< 0.001*	ns
	Test (25)	3.76 ± 0.69	–	3.34 ± 0.67	3.29 ± 0.55	–	–0.43 ± 0.33	–0.48 ± 0.34	< 0.001*	ns
CAL (mm)	Control (27)	5.00 ± 1.04	–	4.70 ± 1.17	4.60 ± 1.23	–	–0.25 ± 0.45	–0.40 ± 0.68	< 0.001*	ns
	Test (25)	4.75 ± 1.00	–	4.49 ± 1.02	4.46 ± 1.00	–	–0.25 ± 0.44	–0.28 ± 0.54	< 0.001*	ns
REC (mm)	Control (27)	1.03 ± 0.63	–	1.30 ± 0.90	1.30 ± 0.85	–	+ 0.33 ± 0.36	+ 0.27 ± 0.41	< 0.05*	ns
	Test (25)	0.97 ± 0.63	–	1.15 ± 0.70	1.16 ± 0.69	–	+ 0.19 ± 0.39	+ 0.20 ± 0.47	ns	ns
FMPS (%)	Control (27)	84.44 ± 20.76	–	56.21 ± 21.64	63.38 ± 22.48	–	–27.54 ± 23.16	–21.06 ± 20.94	< 0.05*	ns
	Test (25)	78.47 ± 25.66	–	55.60 ± 24.97	55.37 ± 29.36	–	–21.00 ± 33.42	–21.23 ± 35.62	ns	ns
Turesky Index	Control (27)	1.93 ± 0.73	1.47 ± 0.48	1.20 ± 0.50	1.40 ± 0.62	–0.46 ± 0.73	–0.73 ± 0.63	–0.47 ± 0.80	< 0.05*	ns
	Test (25)	2.13 ± 0.60	1.69 ± 0.52	1.46 ± 0.58	1.52 ± 0.74	–0.45 ± 0.48	–0.67 ± 0.67	–0.63 ± 0.45	< 0.05*	ns
aMMP-8 (ng/mL)	Control (27)	67.04 ± 103.37	49.00 ± 73.24	27.19 ± 24.73	25.92 ± 27.57	–18.04 ± 41.92	–24.66 ± 62.57	–43.32 ± 106.41	ns	ns
	Test (25)	35.72 ± 31.13	54.68 ± 80.53	25.09 ± 26.37	29.17 ± 29.05	+ 18.96 ± 78.47	–17.66 ± 46.58	–3.63 ± 28.06	ns	ns

Abbreviations: *BoP* bleeding on probing, *PD* probing depth, *CAL* clinical attachment loss, *REC* gingival recession, *FMPS* Full Mouth Plaque Score, *aMMP-8* active matrix metalloproteinase-8.

*Statistically significant within-group change (*p* < 0.05). ns = not significant.

Values are mean ± standard deviation (SD). Within-group comparisons tested with Friedman’s Two-Way Analysis; between-group comparisons with Mann–Whitney U Test.

### Secondary outcomes

#### Probing Depth (PD)

Both groups showed significant reductions in mean probing depth (PD) from baseline to 6 months (*p* < 0.001; Table 2). Baseline PD values were comparable. By 4 months, the mean PD was 3.39 ± 0.48 mm (control) and 3.34 ± 0.67 mm (test), with similar values at 6 months (3.35 ± 0.54 mm and 3.29 ± 0.55 mm, respectively; Table 2). No significant between-group differences were found at any time point (*p* > 0.05; Mann–Whitney U test).

At the site level, the percentage of sites with PD > 5 mm showed minor changes without significant differences (4-month: control 49.6% ± 25%, test 50.4% ± 23%; 6-month: control 54.1% ± 27%, test 45.9% ± 23%).

#### Clinical Attachment Loss (CAL)

Significant CAL improvements were observed within both groups over 6 months (*p* < 0.001; Table 2). Baseline CAL was 5.00 ± 1.04 mm in the control group and 4.75 ± 1.00 mm in the test group (*p* = 0.314). Gains remained stable between 4 and 6 months (control: *p* = 0.413; test: *p* = 0.546). No significant differences were found between groups at any interval (*p* > 0.05), indicating that adjunctive dual-light aPDT offered no additional CAL benefit over NSPT alone.

#### aMMP-8 Levels

Active MMP-8 (aMMP-8) levels declined progressively in both groups. However, none of the intra- or inter-group changes reached statistical significance (Supplementary Table S2).

#### Turesky plaque index and Full-Mouth Plaque Score (FMPS)

Plaque accumulation decreased over time in both groups, as measured by the Turesky Index (Table 2). Baseline scores were comparable (control: 1.93 ± 0.73; test: 2.13 ± 0.60; *p* = 0.161). At 2 weeks, scores significantly declined (control: 1.47 ± 0.48, *p* = 0.013; test: 1.69 ± 0.52, *p* = 0.006), with improvements sustained at 4 months. At 6 months, scores slightly increased (control: 1.40 ± 0.62; test: 1.52 ± 0.74), but the 4–6-month change was not significant (*p* > 0.05). Overall, reductions from baseline remained significant (control: Δ = –0.47 ± 0.80, p = 0.013; test: Δ = –0.63 ± 0.45, *p* < 0.001), with between-group differences remaining non-significant, though nearing significance at 6 months (*p* = 0.054).

FMPS also declined in both groups. Baseline values were similar (control: 84.44 ± 20.76%; test: 78.47 ± 25.66%; *p* = 0.680). At 4 months, FMPS fell to 56.21 ± 21.64% (control, *p* = 0.006) and 55.60 ± 24.97% (test, *p* = 1.000). At 6 months, values were 63.38 ± 22.48% (control) and 55.37 ± 29.36% (test). The 6-month reduction remained significant only in the control group (Δ = –21.06 ± 20.94%, *p* = 0.009). No significant between-group differences were observed at any time point (*p* > 0.05).

#### Compliance to intervention– supplementary Fig. 4

Compliance to dual-light aPDT was high, with a mean usage of 77.7% (range: 11.7%–100%). “Compliant” users (≥ 70% usage) comprised 72% of the test group. Baseline clinical parameters did not differ significantly among compliant, non-compliant, and control groups (*p* > 0.05; Kruskal–Wallis).

Compliant participants demonstrated significant improvements over time in PD, CAL, BoP, and plaque indices (*p* < 0.05; Friedman’s test), with the Turesky Index showing the most improvement. Non-compliant participants showed milder gains, and FMPS increased over time.

Despite favorable within-group trends, no significant between-group differences were detected at any time point (*p* > 0.05).

### Other confounding factors

Patients were stratified by oral hygiene using an FMPS threshold of 40%. In the control group, individuals with FMPS ≤ 40% consistently showed significantly better outcomes.

Mean PD was significantly lower in the FMPS ≤ 40% subgroup at baseline (*p* = 0.015), 4 months (*p* = 0.019), and 6 months (*p* = 0.005). CAL was also more favorable in this group, with significant differences at all time points (baseline: *p* = 0.037; 4 months: *p* = 0.049; 6 months: *p* = 0.024).

Notably, the association between lower FMPS and better PD/CAL outcomes was also evident in the control group at baseline, 4 months, and 6 months, indicating that plaque control, independent of allocation, was the main driver of clinical improvement [22].

Stratification by smoking exposure (pack-years) revealed no significant differences in PD, CAL, or other clinical parameters across light (0.1–7.4), moderate (7.5–20), and heavy (> 20) smokers (*p* > 0.05), based on Susin et al. [27] [27].

### Patient-reported outcomes (PROMs) and adverse effects

Patients in the test group completed the Oral Health Quality of Life (OHQL) questionnaire to evaluate their experience with dual-light aPDT. Most participants (84%) reported using the device as instructed, and 88% found it easy to operate. However, 44% experienced mild discomfort, including sensations of warmth, excess saliva, or a gag reflex, leading to discontinuation in 16% of cases.

Perceived benefits included improved oral hygiene (80%), reduced halitosis (92%), and decreased gum bleeding (88%). Overall, 84% would recommend the device, though only 44% expressed willingness to purchase it. One device-related adverse event was recorded—a minor papilla burn caused by mechanical damage from biting. The injury entirely resolved within two weeks following device discontinuation.

## Discussion

This randomized clinical trial evaluated the adjunctive effect of home-applied dual-light aPDT in smokers with stage III/IV periodontitis. Our main findings were that both groups improved significantly in BoP, PD, and CAL, but no statistically significant intergroup differences were observed. These results are consistent with previous studies showing limited additional benefits of adjunctive aPDT when combined with NSPT [5, 7]. In the following sections, we compare our findings with the literature, highlight their clinical implications, and discuss study limitations.

Our findings show dual-light aPDT produced some short-term benefits, particularly in reducing bleeding on probing (BoP) at two weeks (− 33.60% vs. − 23.94% in controls, *p* = 0.056), but this effect did not persist as a significant advantage at later follow-ups. Both groups maintained intra-group reductions in BoP over six months, affirming the efficacy of NSPT and supportive oral hygiene [28]. However, no intergroup differences were significant beyond the initial phase, suggesting the anti-inflammatory effects of dual-light aPDT may be transient.

Reductions in probing depth (PD) and improvements in clinical attachment loss (CAL) were statistically significant within both groups, in line with previous studies emphasizing NSPT as the cornerstone of periodontal care [28]. The test group showed slightly better CAL outcomes at 4 months (*p* = 0.003), but these were not significant as intergroup differences. This transient improvement may reflect the photobiomodulatory effects of aPDT on tissue healing and collagen remodeling [29–31].

When stratified by plaque control (FMPS ≤ 40% vs. > 40%), participants with lower FMPS exhibited more favorable PD, CAL, and BoP outcomes in both test and control groups. Therefore, our data do not support the conclusion that adjunctive dual-light aPDT compensates for inconsistent hygiene; rather, clinical improvements were primarily associated with plaque control, consistent with established literature underscoring effective self-performed plaque removal—and supportive care—as determinants of periodontal stability [32]. Any apparent differences among adherent device users should be interpreted as hypothesis-generating, as between-group effects were not statistically significant.

Levels of active MMP-8—a biomarker of tissue inflammation—showed inconsistent trends and high interpatient variability. An initial spike in the test group at two weeks may reflect an inflammatory clearing response or tissue remodeling due to subgingival instrumentation, followed by normalization. However, these fluctuations were not statistically significant, highlighting the complex interplay between host response and treatment [33]. Future studies incorporating multiplex biomarker panels could shed light on the molecular mechanisms at play.

A critical insight from our analysis is that patient compliance with daily dual-light aPDT strongly influenced clinical outcomes. Although compliance was not an explicit study objective, we observed that higher compliance to device use was associated with more favorable clinical trends. This highlights the potential importance of patient-friendly adjunctive devices in supporting treatment adherence, which may enhance overall periodontal outcomes.

Patient-reported data revealed high satisfaction with the device: 88% reported easier hygiene, and 92% experienced a reduction in halitosis. However, half reported minor discomfort (e.g., warmth, salivation, gag reflex), and 16% discontinued use. Cost was a barrier for many, with only 44% expressing willingness to purchase the device. Still, the safety profile was favorable, with only one minor adverse event reported.

Despite structured reinforcement, plaque control remained suboptimal in both groups, evident in FMPS scores. Improvements were noted in the Turesky Index, likely due to easier access to anterior buccal surfaces. This reflects the broader challenge of behavior change in smokers, who often show lower compliance with oral hygiene protocols [34].

Emerging evidence suggests that near-infrared light may support mitochondrial energy production by activating cytochrome c-oxidase [35]. However, nicotine impairs mitochondrial function, potentially attenuating these benefits. This raises the possibility that smoking may blunt the cellular benefits of photo biomodulation, an important consideration when applying aPDT in smoker populations [21]. Thus, integrating smoking cessation strategies alongside aPDT may enhance outcomes, as recommended in EFP guidelines [22].

In this study, smoking cessation motivation relied primarily on positive reinforcement, which may have limited impact compared with structured behavioral interventions. Notably, none of the participants expressed interest in smoking cessation despite repeated encouragement. This observation is consistent with national data showing persistently high smoking prevalence in Greece (European Commission, 2024; WHO, 2024). While smoking cessation was not a primary aim of this trial, this lack of motivation highlights a broader public health challenge: periodontal interventions in smokers may be hindered by low readiness to quit. The relevance of these national trends is that they contextualize the difficulty of achieving behavioral change in our study population, underlining the importance of integrating structured cessation support into future periodontal trials rather than drawing direct conclusions on smoking behavior from our data. Embedding systematic behavioral support within periodontal therapy is consistent with EFP S3 guidelines and public-health recommendations, and may enhance both cessation outcomes and periodontal healing.

This study presented certain limitations. The absence of a placebo-controlled group makes it difficult to separate the effects of light application from patient behavior. The sample size was modest, and dropouts reduced statistical power. One methodological limitation was the inability to implement participant or investigator blinding. The physical properties of the investigational device—emitting visible light and mild heat—made it impractical to create a credible sham. Smoking intensity and compliance were self-reported, which could affect data accuracy. Moreover, an important limitation of our study design is the absence of structured oral hygiene reinstruction or supragingival cleaning between the subgingival debridement sessions and 4 months (3-month time interval). Interdental space changes that occur after subgingival instrumentation often necessitate earlier reinforcement, and shorter recall intervals are typically recommended to optimize patient compliance and oral hygiene performance. Our decision to set a longer reevaluation period was intended to reduce professional influence on outcomes and to observe patient-driven behavior in a real-world context. Nevertheless, this may have contributed to the suboptimal plaque control observed in both groups and should be addressed in future studies. Another limitation concerns the choice of BoP as the primary outcome. BoP is a widely used indicator of gingival inflammation, but its diagnostic sensitivity is reduced in smokers due to nicotine-induced vasoconstriction and impaired gingival blood flow. As a result, BoP underestimates inflammatory activity in this population [36, 1]. Probing depth (PD), on the other hand, is less influenced by smoking and may represent a more reliable primary outcome measure for intervention studies in smokers. Future RCTs on periodontal therapies in smokers may benefit from prioritizing PD, complemented by CAL, as primary clinical endpoints. Lastly, the 6-month follow-up may not fully reflect long-term outcomes.

Future studies should address these gaps by implementing placebo-controlled, examiner-blinded, and longer-term trials, ideally stratified by smoking intensity, hygiene behavior, and host susceptibility. Investigating mitochondrial modulation in smokers using near-infrared light could offer novel therapeutic insights. In parallel, improving the design, comfort, and affordability of light-based devices will be essential for maximizing real-world compliance and accessibility.

## Conclusion

In smokers with advanced periodontitis, adjunctive home use of dual-light aPDT did not provide statistically significant improvements over NSPT alone in clinical parameters such as BoP, PD, or CAL. However, high compliance with device use was associated with favorable trends in clinical outcomes, suggesting that compliance is critical to realizing the potential benefits of photodynamic therapy.

## Supplementary Information

Below is the link to the electronic supplementary material.

Supplementary file1 (DOCX 3112 KB)

## Data Availability

The datasets generated and/or analyzed during the current study are available from the corresponding author upon reasonable request.
