# Supplementary material for: Regular home use of dual-light photodynamic therapy as an adjunct to non-surgical periodontal treatment in smokers: a single-center randomized controlled clinical trial
Source: Clin Oral Investig. 2025 Nov 6;29(12):553. doi: 10.1007/s00784-025-06600-1 (PMC12592251; doi:10.1007/s00784-025-06600-1)
Supplement: Supplementary file 1 — Supplementary file1 (DOCX 3112 KB) [file 784_2025_6600_MOESM1_ESM.docx]

**Suppl. Materials**

### Suppl Table 1: CONSORT 2010 Checklist for RCTs

| **Section / Topic** | **ItemNo.** | **Checklist Item** | **Reported on Page No.** |
| --- | --- | --- | --- |
| **Title and Abstract** | 1a | Identification as a randomized trial in the title | Titlepage |
|  | 1b | Structured summary of trial design, methods, results, and conclusions | Abstract |
| **Introduction** | 2a | Scientific background and explanation of rationale | Introduction |
|  | 2b | Specificobjectivesorhypotheses | End of Introduction |
| **Methods** | 3a | Description of trial design (e.g., parallel, allocation ratio) | Methods → Study design |
|  | 3b | Important changes to methods after trial commencement (with reasons) | Notapplicable |
|  | 4a | Eligibilitycriteria for participants | Methods → Eligibility |
|  | 4b | Settings and locations where the data were collected | Methods → Recruitment |
|  | 5 | Interventions for each group with sufficient details to allow replication | Methods → NSPT and aPDT |
|  | 6a | Defined pre-specified primary and secondary outcome measures | Methods → Clinicaloutcomes |
|  | 6b | Changes to trial outcomes after commencement | Notapplicable |
|  | 7a | Samplesizedetermination | Methods → Samplesize |
|  | 7b | Interim analyses and stopping guidelines | Notapplicable |
|  | 8a | Method used to generate the random allocation sequence | Methods → Randomization |
|  | 8b | Type of randomization and details of any restriction | Simplerandomization |
|  | 9 | Allocationconcealmentmechanism | Opaqueenvelopes |
|  | 10 | Implementation: Who generated the sequence, enrolled, and assigned participants | Described (D.S. did randomization) |
|  | 11a | Blinding (who was blinded and how) | Notblinded |
|  | 11b | Description of the similarity of interventions | N/A (interventionvisible) |
|  | 12a | Statistical methods used to compare groups for primary/secondary outcomes | Methods → Statistics |
|  | 12b | Methods for additional analyses (e.g., subgroup, adjusted) | Described for compliance and FMPS subgroups |
| **Results** | 13a | Participant flow: numbers assigned, treated, analyzed | Results + CONSORT Figure |
|  | 13b | Losses and exclusions with reasons | Results → Flowparagraph |
|  | 14a | Dates defining recruitment and follow-up | Jan 2023 – June 2024 |
|  | 14b | Why trial ended or was stopped | Notapplicable |
|  | 15 | Baseline demographic and clinical characteristics | Table 1 |
|  | 16 | Numbers analyzed per group, with reason | Results + CONSORT figure |
|  | 17a | Outcomes and effect sizes for each primary and secondary outcome | Tables 2–6, Supp. Tables |
|  | 17b | Binary outcomes: absolute and relative effect sizes | Notapplicable |
|  | 18 | Ancillary analyses (e.g., compliance, FMPS stratification) | Compliancesection |
|  | 19 | Harmsorunintendedeffects | Reported (1 adverseevent) |
| **Discussion** | 20 | Triallimitations | Discussionsection |
|  | 21 | Generalizability (externalvalidity) | Discussion (smokers) |
|  | 22 | Interpretation consistent with results, benefits, harms, limitations | Discussion and Conclusion |
| **Other Information** | 23 | Trialregistration | ClinicalTrials.gov: NCT05962801 |
|  | 24 | Protocolavailability | Notexplicitlyreported |
|  | 25 | Sources of funding and role of funders | Fundingsection: Nofunding |

**Suppl. Figure 1.**Lumoral® starter kit. (A) The kit includes the Lumoral® device and the effervescent Lumorinse® tablets (*) to be dissolved in 30 mL of water with a measuring cup (**). A power source (***) for the light applicator. (B). The mouthpiece (*) is composed of 48 LED components (**) assembled to provide simultaneous light on both the maxillary and mandibular dental arches. Each LED component emits 405 nm and 810 nm light simultaneously. A push of the control button provides a treatment time of 10 min (***). When dissolved, the ICG in the Lumorinse® tablet appears green.


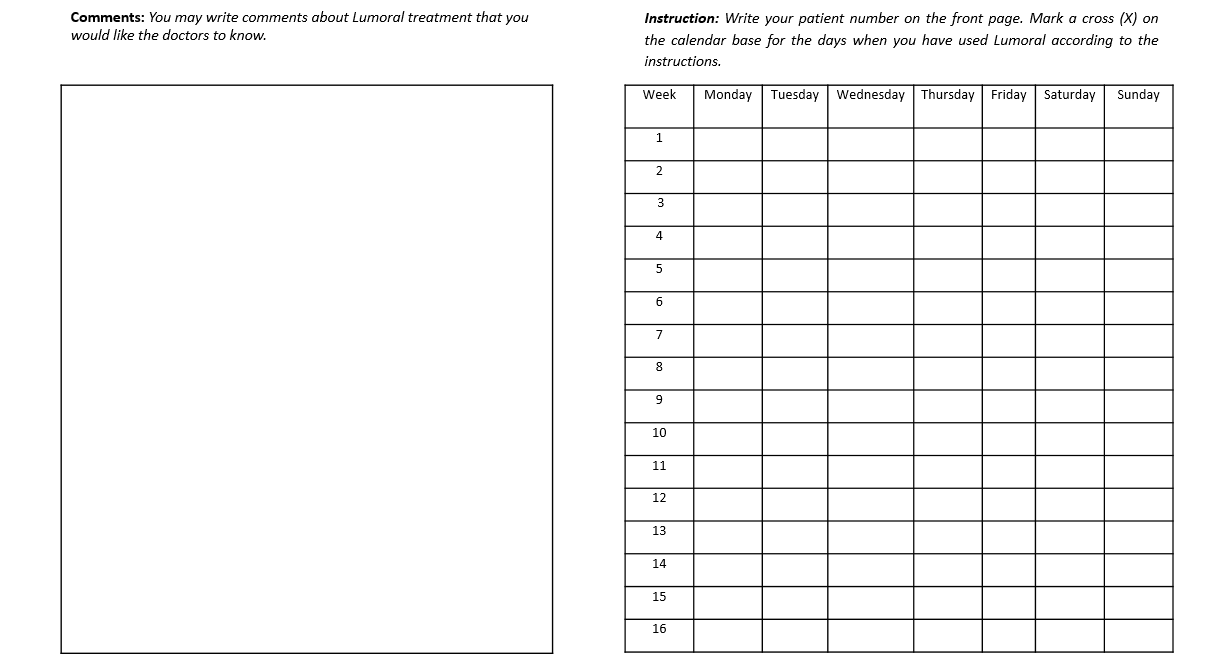


**Suppl. Figure 2**. Patient's diary regarding Lumoral® use


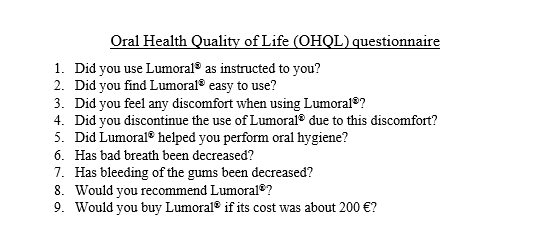


**Figure 3 Suppl.** Oral Health Quality of Life (OHQL) questionnaire distributed to test group patients


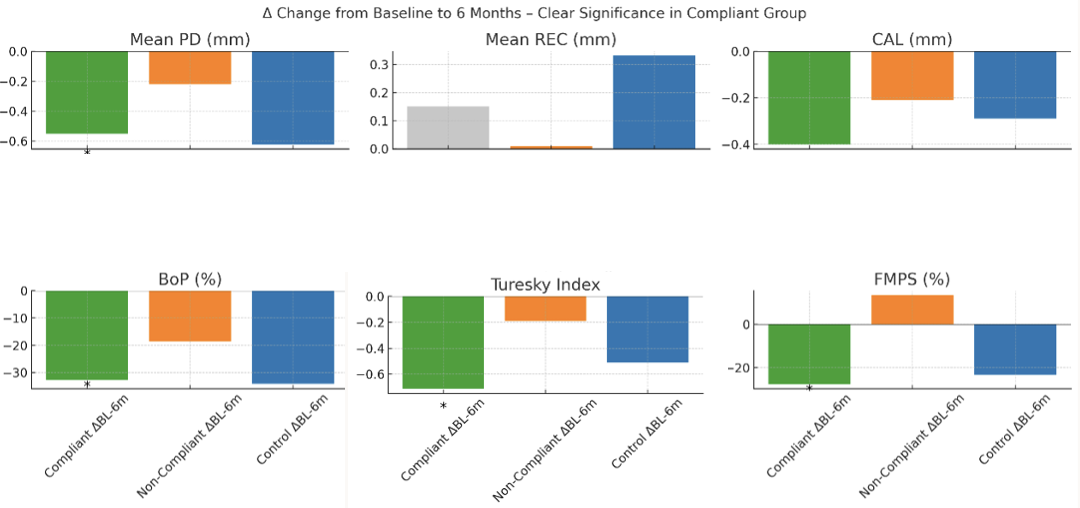


**Suppl. Figure 4:** Supplementary Figure 4. Compliance to Intervention and Clinical Outcomes

**Supplementary Table S1. Mean aMMP-8 Levels (ng/mL), Temporal Changes, and Statistical Comparisons.**

Active MMP-8 (aMMP-8), a host-derived biomarker of periodontal inflammation, decreased over the study period in both groups. While early reductions were slightly more pronounced in the control group, no statistically significant differences were found between or within groups at any time point. These findings suggest that NSPT effectively reduces host inflammatory burden, but adjunctive dual-light aPDT did not confer additional measurable benefit in aMMP-8 modulation in this cohort.

| Parameter | Group (n) | Baseline | 2 Weeks | 4 Months | 6 Months | Δ 2w–BL | Δ 4m–2w | Δ 6m–4m | Δ 6m–BL | p(4m–2w) | p(6m–4m) | p(4m–BL) | p(2w–BL) | p(6m–BL) | p(2w–6m) |
| --- | --- | --- | --- | --- | --- | --- | --- | --- | --- | --- | --- | --- | --- | --- | --- |
| Mean aMMP-8 ± SD (ng/mL) | Control (27) | 67.04 ± 103.37 | 49.00 ± 73.24 | 27.19 ± 24.73 | 25.92 ± 27.57 | –18.04 ± 41.92 | –-24.66 ± 62.57 | –0.85 ± 24.64 | –43.32 ± 106.41 | 1.000 | 1.000 | 0.302 | 1.000 | 0.202 | 1.000 |
|  | Test (25) | 35.72 ± 31.13 | 54.68 ± 80.53 | 25.09 ± 26.37 | 29.17 ± 29.05 | +18.96 ± 78.47 | –17.66 ± 46.58 | +4.25 ± 33.00 | –3.63 ± 28.06 | 1.000 | 1.000 | 1.000 | 1.000 | 1.000 | 1.000 |
|  | Total (52) | 52.00 ± 78.44 | 51.73 ± 76.13 | 26.18 ± 25.28 | 27.48 ± 28.04 | –0.26 ± 64.36 | –21.31 ± 55.02 | +1.59 ± 28.72 | –24.27 ± 80.92 | – | – | – | – | – | – |

Between-Group Comparisons (p-value):
Baseline: 0.660 | 2 Weeks: 0.557 | 4 Months: 0.530 | 6 Months: 0.808
Δ 2w–BL: 0.200 | Δ 4m–2w: 0.992 | Δ 6m–4m: 0.259 | Δ 6m–BL: 0.264

Abbreviations: aMMP-8: active matrix metalloproteinase-8; SD: Standard Deviation.
Tests: Related Samples Friedman’s Two-Way Analysis (within-group); Mann–Whitney U Test (between-group).

**Supplementary Table S2. Number of pockets >4mm per patient and group.**

|  |  | Baseline | 4 month re-evaluation | 6 month re-evaluation |
| --- | --- | --- | --- | --- |
|  | Patient no |  |  |  |
| Test | 1 | 61/168 | 47/162 | 39/156 |
|  | 3 | 40/168 | 10/168 | 24/168 |
|  | 6 | 22/156 | 12/156 | 26/156 |
|  | 7 | 19/114 | 22/114 | 17/114 |
|  | 12 | 6/144 | 3/144 | 4/144 |
|  | 16 | 32/132 | 30/126 | 23/114 |
|  | 18 | 54/126 | 25/114 | 25/114 |
|  | 20 | 86/114 | 68/114 | 51/102 |
|  | 21 | 87/150 | 58/150 | 48/150 |
|  | 25 | 43/168 | 40/168 | 56/168 |
|  | 27 | 23/102 | 21/102 | 14/102 |
|  | 30 | 18/168 | 4/168 | 1/168 |
|  | 33 | 25/174 | 3/174 | 7/174 |
|  | 35 | 8/108 | 2/96 | 0/96 |
|  | 37 | 59/174 | 53/168 | 37/168 |
|  | 40 | 100/156 | 77/156 | 26/156 |
|  | 42 | 34/138 | 16/138 | 20/138 |
|  | 43 | 17/144 | 9/138 | 7/138 |
|  | 45 | 25/144 | 5/144 | 9/144 |
|  | 56 | 15/120 | 1/120 | 8/120 |
|  | 58 | 35/138 | 13/132 | 14/132 |
|  | 59 | 50/147 | 15/138 | 20/138 |
| Control | 2 | 39/108 | 15/108 | 16/102 |
|  | 5 | 102/150 | 56/144 | 51/144 |
|  | 8 | 57/132 | 19/132 | 13/132 |
|  | 10 | 24/132 | 8/132 | 11/132 |
|  | 11 | 31/156 | 30/156 | 31/156 |
|  | 19 | 57/96 | 16/96 | 17/96 |
|  | 22 | 20/192 | 18/192 | 12/192 |
|  | 24 | 56/162 | 18/156 | 17/156 |
|  | 26 | 35/108 | 25/108 | 31/108 |
|  | 28 | 41/114 | 51/114 | 33/102 |
|  | 29 | 74/168 | 67/168 | 38/168 |
|  | 32 | 13/120 | 6/120 | 3/120 |
|  | 36 | 80/162 | 24/150 | 27/150 |
|  | 38 | 46/144 | 19/138 | 14/138 |
|  | 39 | 50/132 | 50/132 | 67/132 |
|  | 41 | 26/102 | 17/90 | 12/90 |
|  | 44 | 12/144 | 5/144 | 2/144 |
|  | 46 | 27/150 | 14/150 | 17/150 |
|  | 49 | 22/90 | 8/90 | 7/90 |
|  | 51 | 47/138 | 18/138 | 27/138 |
|  | 53 | 49/108 | 7/108 | 10/108 |
|  | 55 | 42/114 | 27/108 | 23/114 |
|  | 57 | 10/162 | 4/162 | 3/162 |
|  | 60 | 62/156 | 40/156 | 41/156 |
